# 1412. Bioburden Reduction: Introduction of a novel skin antiseptic solution

**DOI:** 10.1093/ofid/ofad500.1249

**Published:** 2023-11-27

**Authors:** Diana Fernández-Rodríguez, Jeongeun Cho, Emanuele Chisari, Javad Parvizi

**Affiliations:** MD/PhD Plan de Estudios Combinados en Medicina (PECEM), Mexico City, Distrito Federal, Mexico; Rothman Orthopaedic Institute, Philadelphia, Pennsylvania; Rothman Orthopaedic Institute, Philadelphia, Pennsylvania; Rothman Orthopaedic Institute, Philadelphia, Pennsylvania

## Abstract

**Background:**

Prevention of healthcare-associated infections (HAI), like surgical site infections (SSIs), has become a top priority for the medical community. Reduction of the bioburden on the skin is a proven strategy that minimizes SSI. All the currently available antiseptic skin products, for skin decolonization, are chlorhexidine gluconate (CHG) based. The rise in resistance of organisms to CHG, increase in reported cases of CHG hypersensitivity, and the lack of activity of CHG against some common pathogens, prompted us to design a novel skin antiseptic solution.

**Methods:**

The antiseptic solution tested contained benzalkonium chloride (BZK) at 0.129% as the main ingredient. *In vitro* time to kill assay was conducted according to ASTM protocol E2315-16. We reported CFU/ml at 3 different timepoints after exposure (30s, 60s, and 120s). The baseline inoculum for all the 16 reference strains was 10^5^ CFU/ml. We included 8 Gram-positive, 5 Gram-negative, 2 anaerobes, and 1 fungal species. Experiments were performed two times in triplicate.

**Results:**

At 30s of exposure, only 2 out of the 16 strains tested (12.5%) remained viable: *Staphylococcus aureus* (ATCC 29213) and *Candida albicans* (ATCC 18804). In fact, we observed a 2-log reduction in *S. aureus* (83.3 ± 98.3 CFU/ml) and almost 1-log reduction in *C. albicans* (1 867 ± 417.9 CFU/ml), from the baseline inoculum. After 30s of exposure, all bacterial strains were killed by the testing solution; however, *C. albicans* was still found at 120s of exposure (666.7 ± 535.4 CFU/ml).

In vitro efficacy of a CHG-based and a BZK-based solution at 3 different timepoints (30 seconds, 60 seconds, and 120 seconds).

**Figure:**
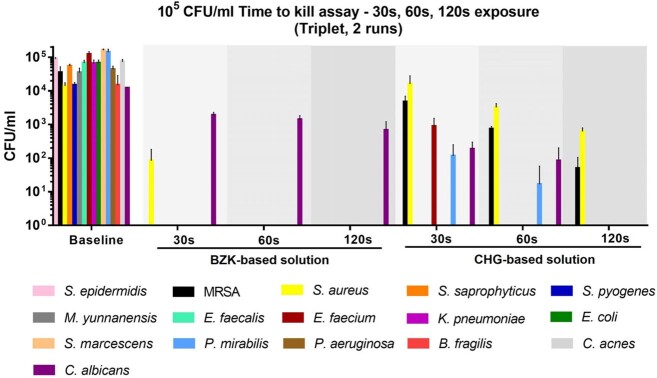

**Conclusion:**

The BZK-based solution was effective against all Gram-positive, Gram-negative, and anaerobic bacterial strains, after 30s of exposure. The rapid action of BZK, together with its proven safety, makes it a promising product for skin decolonization in the future.

**Disclosures:**

**Javad Parvizi, MD, FRCS**, 3M: Grant/Research Support|Acumed, LLC: Stocks/Bonds|Aesculap: Grant/Research Support|Alphaeon: Stocks/Bonds|AO Spine: Stocks/Bonds|Becton Dickenson: Advisor/Consultant|Biomet: Grant/Research Support|Cardinal Health: Advisor/Consultant|Cempra: Grant/Research Support|CeramTec: Grant/Research Support|Ceribell: Stocks/Bonds|Coracoid: Stocks/Bonds|Corentec: Advisor/Consultant|Datatrace: Grant/Research Support|DePuy: Grant/Research Support|Elsevier: Grant/Research Support|Elute: Stocks/Bonds|Ethicon: Advisor/Consultant|Hip Innovation Technology: Stocks/Bonds|Illuminus: Stocks/Bonds|Integra: Grant/Research Support|Intellijoint: Stocks/Bonds|Jaypee Publishers: Grant/Research Support|KCI / 3M (Acelity): Advisor/Consultant|Lima: Grant/Research Support|MicroGenDx: Advisor/Consultant|Molecular Surface Technologies: Stocks/Bonds|Myoscience: Grant/Research Support|Nanooxygenic: Stocks/Bonds|National Institutes of Health (NIAMS & NICHD): Grant/Research Support|NDRI: Grant/Research Support|Novartis: Grant/Research Support|OREF: Grant/Research Support|Orthospace: Grant/Research Support|Osteal: Stocks/Bonds|Parvizi Surgical Innovations and Subsidiaries: Stocks/Bonds|Peptilogic: Stocks/Bonds|Peptilogics: Advisor/Consultant|Pfizer: Grant/Research Support|PRN-Veterinary: Grant/Research Support|Rotation Medical: Grant/Research Support|Simplify Medical: Grant/Research Support|SLACK Incorporated: Grant/Research Support|Smith & Nephew: Grant/Research Support|Sonata: Stocks/Bonds|Stelkast: Grant/Research Support|Stryker: Grant/Research Support|Synthes: Grant/Research Support|Tenor: Advisor/Consultant|TissueGene: Grant/Research Support|Tornier: Grant/Research Support|Wolters Kluwer Health - Lippincott Williams & Wilkins: Grant/Research Support|Zimmer Biomet: Advisor/Consultant|Zimmer Biomet: Grant/Research Support

